# Breastfeeding Practices in the United Arab Emirates: Prenatal Intentions and Postnatal Outcomes

**DOI:** 10.3390/nu14040806

**Published:** 2022-02-14

**Authors:** Zainab Taha, Malin Garemo, Farid El Ktaibi, Joy Nanda

**Affiliations:** 1 Department of Health Sciences, Zayed University, Abu Dhabi P.O. Box 144534, United Arab Emirates; malingaremo@gmail.com; 2 Department of Mathematics and Statistics, Zayed University, Abu Dhabi P.O. Box 144534, United Arab Emirates; farid.elktaibi@zu.ac.ae; 3 The Johns Hopkins Medical Institutions, Baltimore, MD 21206, USA; joypnanda@gmail.com

**Keywords:** breastfeeding intention, breastfeeding practices, knowledge, support, duration, United Arab Emirates

## Abstract

Breastfeeding provides the ideal nutrition in infancy, and its benefits extend to the health of mothers. Psychosocial factors such as the intention to breastfeed, self-efficacy, or maternal confidence to breastfeed have been shown to impact breastfeeding outcomes in other communities. The aim of this study was to assess the potential associations between mothers’ prenatal intention to breastfeed and post-delivery breastfeeding practices. A cross-sectional study was conducted from March to September 2017. Emirati and non-Emirati mothers with children below the age of 2 were recruited from maternal and child health centers in various geographical areas in Abu Dhabi Capital district, United Arab Emirates. The variables (mothers’ intention to breastfeed, breastfeeding knowledge, support from family and health care professionals, and initiation and duration of breastfeeding) were collected by research assistants during an in-person interview, using a structured questionnaire. A total of 1799 mothers participated in this study. Mothers’ prenatal intention to breastfeed was significantly associated with breastfeeding initiation (*p* < 0.001) and length of exclusive breastfeeding (*p* = 0.006). Furthermore, intention to breastfeed during early pregnancy showed a strong association (*p* < 0.001) with mothers who had exclusively breast fed for more than three months. In addition, knowledge on the benefits of breastfeeding and getting support from relatives and non-relatives demonstrated significant relationship with a longer period of exclusive breastfeeding (*p* < 0.01) In total, mothers in the study reported receiving almost four thousand advice about breastfeeding, of which 3869 (97%) were encouraging to our others in the study. Our findings on prenatal intentions, knowledge and network support on exclusive breastfeeding indicates the importance of including breastfeeding knowledge and support as critical topics during prenatal education, not only to the mothers but also to close network members who seek a healthy pregnancy outcome.

## 1. Introduction

Breastfeeding provides the ideal method of feeding during infancy, offering adequate nutrition as well as immunological and developmental advantages [1]. Mothers benefit from breastfeeding by having a reduced risk of post-delivery complications, postpartum depression, and certain diseases later in life [2,3]. For both mothers and infants, breastfeeding has been shown to support bonding and emotional interaction [4]. 

Decisions on methods of feeding infants, and breastfeeding practices are influenced by a wide range of individual, cultural, and socioeconomic factors [5,6,7,8,9]. Additionally, psychosocial factors such as the intention to breastfeed, self-efficacy, or maternal confidence to breastfeed have also been shown to impact breastfeeding outcomes [10]. Other less studied factors such as assisted reproduction therapy, emotional health, previous breastfeeding experiences, and mental health issues may also affect breastfeeding habits [11]. Prenatal intention to breastfeed has been shown to influence breastfeeding initiation and duration [12,13,14]. A longitudinal study showed that among women with a prenatal intention to breastfeed, 96.6% initiated breastfeeding compared to 4.4% among those with an intention to bottlefeed [14]. Similar to others, the same study also showed that at six months postpartum, the mean duration of breastfeeding for women intending to breastfeed for at least five months was 4.4 months, compared to 2.5 months among women with a prenatal intention to breastfeed for one month [14,15].

Besides intention, knowledge about breastfeeding benefits is another important factor influencing breastfeeding practices [6]. Knowledgeable women feel more confident during breastfeeding and have a longer duration of breastfeeding [15]. Furthermore, maternal breastfeeding knowledge has positively impacted the intention to breastfeed, attitudes towards exclusive breastfeeding, and self-efficacy [16]. Another study has shown that more knowledge is significantly associated with breastfeeding initiation and duration [17].

Influence from others, both during pregnancy and post-delivery, can impact breastfeeding outcomes [18,19,20,21]. The influence may include emotional support through encouragement and positive enforcement by family and/or health professionals, education programs on breastfeeding, and surroundings that facilitate breastfeeding [22]. Systematic reviews suggest that prenatal interventions alone or in combination with intra/postpartum components, including both education and interpersonal support delivered not only to the expecting mothers, but also to her close family/spouse are effective in increasing breastfeeding initiation, duration, or exclusivity [23,24]. Furthermore, interventions delivered in a combination of settings, i.e., healthcare centers, homes, and in the community, have a better impact on improving breastfeeding rates [24]. Moreover, emotional and practical support provided by husbands and close relatives have also been positively associated with breastfeeding [19,20,21,25]. In contrast, discouragement from close relatives and friends may negatively impact breastfeeding practices [22,26,27].

As described, prenatal intention, knowledge, and support have been shown to impact breastfeeding practices in other cultures, while data are scarce in the United Arab Emirates (UAE). The UAE has embraced the WHO/UNICEF international infant feeding recommendations [28]. However, despite the known benefits and the efforts by the UAE health authorities, breastfeeding rates in the UAE remain suboptimal [29] compared to the WHO’s target to increase the global rate of exclusive breastfeeding to at least 50% by 2025. Hence, a more detailed understanding of childbearing women’s intention to breastfeed and associations to breastfeeding practices could be beneficial for policymakers in developing effective interventions to improve breastfeeding outcomes in the UAE. The aim was to assess the potential associations between mothers’ prenatal intention to breastfeed and the post-delivery breastfeeding practices. 

## 2. Materials and Methods

### 2.1. Participants and Data Collection

**Figure 1:**
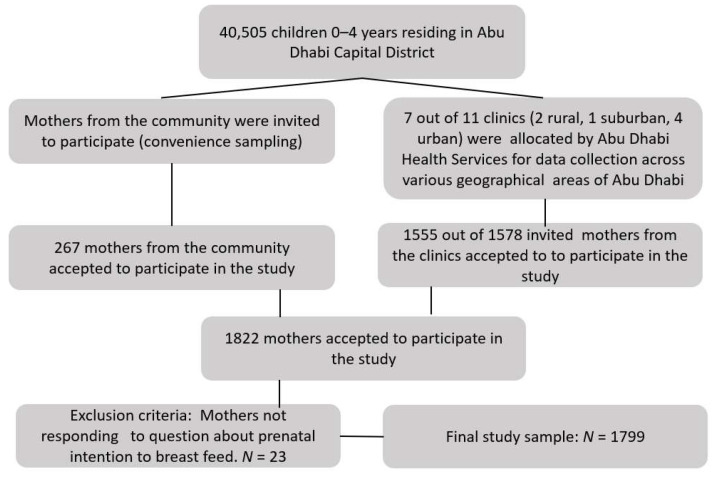
Schematic diagram of the overall recruitment of the study participant, including exclusion criteria.

From different geographical areas in Abu Dhabi Capital district, mothers were recruited from maternal and child health centers and the community. Female research assistants provided oral and written information about the study to Emirati and non-Emirati mothers with children below the age of two. Following the recruitment, consenting mothers who met the inclusion criteria of having answered a specific question regarding their prenatal intention to breastfeed were considered the participating subsample for the current study (Figure 1).

After consent was given, the research assistants followed a structured questionnaire and interviewed the women individually. A detailed description of the design and sampling of the original study from which the current sample was extracted has been published elsewhere [30]. The study was approved (ZU17_006_F) by the Research Ethics Committee at Zayed University, UAE. 

### 2.2. Study Instrument

The questionnaire used in the study was developed for this specific investigation and included questions about family demographics and infant feeding practices. Prior to the main study, a pilot study was conducted using face validity in order to reduce bias. Interviews were chosen as the data collection method to ensure that all women could participate, regardless of literacy level. The questionnaire (Arabic and English) consisted of 57 main questions with several subitems. The questions concerning mothers’ pre- and postnatal intention to breastfeed, breastfeeding knowledge, support from family and health care professionals, as well as the initiation and duration of breastfeeding, were included and analyzed in this study. Data about mothers’ demographics (e.g., education, age, self-rated financial status, nationality, and occupation) were also collected and presented in this paper. Exclusive breastfeeding was defined as the infant being fed only breast milk without any other oral intake except medications and vitamins within the last 24 h. Any breastfeeding was defined as the infant being fed a combination of breastmilk, formula, and/or complementary feeding. 

### 2.3. Statistical Analysis

All statistical analyses were performed using IBM SPSS Statistics Version 27.0 (Endicott, NY, USA). Frequency distributions and percentages generated descriptive statistics to analyze the demographic characteristics of the participants. To examine the association between the dependent variable (breastfeeding initiation or duration) and the independent variables (intention to breastfeed, knowledge about breastfeeding, support received from family members and relatives, and time during pregnancy at which women start to think about breastfeeding), Chi-square tests or Fisher exact tests were conducted wherever appropriate. The significance level was set to *p* < 0.05. Thereafter, multivariate logistic regressions were carried out using the variables that were significant in the bivariate analysis to determine the adjusted odds ratio (aOR) of initiation, knowledge, and support to breastfeeding initiation or duration. The multivariate logistic analysis was used to identify the extent of the impact of the factors with breastfeeding initiation or duration. Consequently, we report the aOR and their 95% confidence intervals (CI) for factors with *p*-values less than 0.05. 

## 3. Results

A total of 1799 mothers participated in this study (Figure 1). As shown in Table 1, more than half of the women were in the age group 25–34 years. Almost all mothers were married; a majority had a university education and reported their financial wellbeing to be very good to excellent. One-third of the participants were Emirati, and the others were either non-Emirati Arab or non-Arab (Table 1).

Table 2 shows the intention, knowledge, and support and their association to breastfeeding initiation and duration. The prenatal intention to breastfeed was significantly associated with both breastfeeding initiation (*p* < 0.001) and exclusive breastfeeding duration (*p* = 0.006) but not with any breastfeeding. Similarly, support received from other people was significantly associated with both breastfeeding initiation (*p* < 0.001) and exclusive breastfeeding duration (*p* = 0.01) but not with any breastfeeding.

Breastfeeding knowledge was significantly associated with exclusive breastfeeding duration (*p* = 0.003) (Table 2). Furthermore, multivariate logistic regression was conducted to determine the factors significantly associated with breastfeeding initiation and duration with aOR and CI. Childbearing women who intended to breastfeed were 9.63 times more likely to initiate breastfeeding (95% CI 5.04–18.39) than those with no intention. In addition, childbearing women who received support and encouragement from family members on breastfeeding were 2.89 times more likely to initiate breastfeeding than women who did not (95% CI 1.50–5.55). Additionally, childbearing women who received support from others were 1.52 times more likely to have a longer duration of breastfeeding than women who did not have any support (95% CI = 1.07–2.15). Childbearing women with medium level and high level of breastfeeding knowledge were 1.29 times and 1.75 times more likely to have a longer duration of EBF than those with low knowledge (95% CI 1.02–1.65) and (95% CI 1.31–2.32), respectively. Childbearing women with medium and high levels of breastfeeding knowledge were 1.47 and 1.50 times more likely to have a longer duration of any breastfeeding than those with low knowledge (95% CI 1.00–2.14) and (aOR = 1.5, 95% CI 1.07–2.06), respectively.

The exclusive breastfeeding duration was significantly impacted by the trimester during which the childbearing women started to think about breastfeeding (*p* < 0.001). As shown in Table 3, a higher proportion of women who intended to breastfeed during the first or second trimester of their pregnancy continued with EBF for ≥ 6 months compared to those who intended to breastfeed during the third trimester. A bigger proportion of childbearing women who intended to breastfeed during the third trimester stopped EBF < 1 month compared to those who intended to breastfeed during the first trimester (Table 3).

The multivariate ordinal regression showed that women who intended to breastfeed during their first trimester were 2.23 times more likely to have a longer duration of exclusive breastfeeding than those without intention to breastfeed (95% CI 1.44–3.44). Women who intended to breastfeed during the second trimester were 1.70 times more likely to breastfeed than those without intention to breastfeed (95% CI = 1.03–2.79).

Table 4 shows who encouraged and discouraged the mothers to breastfeed pre- and post-delivery. In total, there were 3999 instances of advice being offered regarding breastfeeding, of which 3869 (96.7%) were encouraging and 130 (3.3%) were discouraging. Overall, health care professionals, including lactation specialists, offered 2110 pieces (54.0%) of encouraging advice, followed by mothers offering 747 (19.3%), spouses offering 427 (11.0%), mothers-in-law offering 402 (10.4%), and other relatives and friends offering 183 (4.7%).

## 4. Discussion

The current study is one of the first to investigate the association between prenatal breastfeeding intention and breastfeeding outcomes in the UAE. Childbearing women’s prenatal intention to breastfeed was significantly associated with breastfeeding initiation and exclusive breastfeeding duration. Furthermore, support from family and health professionals was significantly associated with breastfeeding initiation and exclusive breastfeeding duration. Despite the long-term adoption of the WHO recommendations for breastfeeding in the UAE, the rates remain suboptimal. In this study, women who started to think about breastfeeding at an early stage of their pregnancies had a higher initiation rate and longer breastfeeding duration, providing some guidance on the importance of early antenatal education, including feeding strategies. Breastfeeding knowledge was significantly associated with exclusive breastfeeding duration further supporting the importance of antenatal education.

Similar to the results in this study, other studies have reported a positive association between prenatal intention to breastfeed and breastfeeding initiation and duration [14,31,32,33]. In this study, an association was found between prenatal intention and EBF but not with partial breastfeeding. Almost everyone with a prenatal intention to breastfeed (97.9%) actually initiated breastfeeding compared to 73.1% among those without prenatal intention confirming findings from the UK where 96.6% of women who intended to breastfeed for at least four months initiated breastfeeding, while only 74.7% of those who intended to breastfeed for less than four weeks actually initiated breastfeeding [14]. Similarly, a study from the US demonstrated that women without intention to breastfeed were 405 times less likely to initiate breastfeeding than to those who intended to breastfeed for 12 months, indicating that a prenatal intention to breastfeed has an impact on both initiation and duration [34]. 

The results in this study indicate that early intention has a positive impact on breastfeeding duration. Others have found that those planning for a short duration stop breastfeeding significantly earlier than those who plan to breastfeed longer [14,31]. Although many studies support the association between prenatal intention and breastfeeding practices, others have found a discrepancy between the intended and actual breastfeeding duration [34,35].

In Saudi Arabia, although the childbearing women were knowledgeable about breastfeeding, less than half of them intended to initiate breastfeeding [36]. In this study, breastfeeding knowledge also did not impact breastfeeding initiation, which stands in contrast to many other studies that found that knowledge predicts breastfeeding initiation [37,38,39]. This may be related to methodological issues inadequately assessing breastfeeding knowledge. On the other hand, a significant association was found between knowledge and exclusive breastfeeding duration, similarly to what has been reported by [8,14,40,41], but the findings are not conclusive, hence this area needs further investigation [42].

As reported by the mothers, and similar to other studies, health professionals and families impacted breastfeeding practices [18,20,22]. Not surprisingly, the health professionals gave the most advice, followed by the participants’ mothers, mothers-in-law, and their spouses, in line with another study in the UAE [43]. Others have reported that paternal support and knowledge about breastfeeding significantly impacts breastfeeding success [44]. A systematic review, including 14 countries, found that all forms of laymen and professional support, especially in combination, increase the duration of breastfeeding, especially exclusive breastfeeding, up to the first six months, which is similar to the findings in this study [45]. Paid maternity leave has been shown to positively impact the rate of mothers attempting to breastfeed [46]. During the time of data collection, the UAE maternity leave for governmental employees was 3 months of paid leave and shorter working hours for the first year after giving birth, but despite this, the breastfeeding rates in the UAE are suboptimal. In order to reach the set target, the current breastfeeding policies may take all of the above findings into account by implementing early and continuous antenatal education, not only of the childbearing women but also close family members [30].

A major strength of the study is that a large sample of mothers, both UAE nationals and expatriates, were recruited from the majority of the centers rendering maternal and health services located in various geographical areas in Abu Dhabi Capital district, as well as from the community, making the sample likely to be representative of Abu Dhabi. Another strength of the study is the attempt to understand women’s breastfeeding perceptions in addition to their actual experiences. 

A limitation of this study, and other similar cross-sectional studies, is related to the accuracy of long-term maternal recall of breastfeeding practices. Although mothers’ knowledge about breastfeeding was assessed briefly, future studies should address the gap related to potential associations between knowledge and practices. In addition, challenges associated with physical or emotional health that may impact breastfeeding intention and outcomes were not included in this study and that can also be seen as a limitation.

## 5. Conclusions

To conclude, it was shown that the vast majority of breastfeeding advice was perceived as encouraging by the mothers. Support towards breastfeeding and the prenatal intention to breastfeed showed positive associations, both with breastfeeding initiation and the exclusive breastfeeding duration. Furthermore, women who started to think about breastfeeding early on in the pregnancy were more likely to have a longer duration of breastfeeding. This indicates the importance of including breastfeeding as a continuous topic during the prenatal education sessions, not only to the mothers but also to the close family members. This can have a positive impact on the breastfeeding intention and thus, on the breastfeeding practices which therefore would work more efficiently towards reaching the WHO goals. 

## Figures and Tables

**Table 1:** Maternal demographic. *N* = 1799.

Characteristics	Frequency	%
Mother’s Age (years)		
17–19	17	0.9
20–24	232	12.9
25–34	1153	64.1
35–51	397	22.1
Mother’s Nationality ^a^		
Emirati	582	32.4
Non Emirati-Arab	606	33.8
Non Emirati-Non Arab	607	33.8
Marital Status ^b^		
Married	1764	98.5
Un-married	26	1.5
Mother’s Education ^c^		
Below High School	76	4.3
High School	338	19
University	1364	76.7
Father’s Education ^d^		
Below High School	38	2.1
High School	202	11.3
University	1551	86.6
Mother’s Employment Status		
Employed	646	35.9
Not Employed	1153	64.1
Family Financial Well Being ^e^ (FFWB)		
Excellent/Very Good	1195	66.6
Good	480	26.8
Fair	106	5.9
Poor/Very Poor	12	0.7
Parity		
1st child	644	35.8
2nd child	535	29.8
3rd child	305	17.0
4th child	312	17.4

^a^ 4 missing data, ^b^ 9 missing data, ^c^ 21 missing data, ^d^ 8 missing data, ^e^ 6 missing data.

**Table 2:** Intention, knowledge, and support and their association to breastfeeding (BF) initiation and duration.

Pre-Delivery	Post-Delivery *N* (%)												
	Breastfeeding Initiation			Exclusive Breastfeeding					Any Breastfeeding				
		No	*p*-Value	<1 Month	1–3 Months	3–6 Months	>6 Months	*p*-Value	<1 Month	1–3 Months	3–6 Months	>6 Months	*p*-Value
Intention to BF													
Yes	1655 (97.9)	36 (2.1)	<0.001	292 (22.7)	388 (30.1)	298 (23.2)	309 (24.0)	0.006	24 (2.4)	110 (11.2)	143 (14.6)	703 (71.7)	0.803
No	79 (73.1)	29 (26.9)		26 (35.6)	19 (26.0)	21 (28.8)	7 (9.6)		1 (1.8)	8 (14.0)	10 (17.5)	38 (66.7)	
Knowledge about BF													
100% correct answers	394 (96.1)	16 (3.9)	0.331	62 (19.6)	90 (28.4)	68 (21.5)	97 (30.6)	0.003	7 (2.7)	26 (9.9)	38 (14.4)	192 (73.0)	0.197
50% correct answers	975 (96.9)	31 (3.1)		173 (23.3)	220 (29.6)	180 (24.2)	170 (22.9)		11 (2.0)	63 (11.4)	72 (13.0)	407 (73.6)	
0% correct answers	365 (95.3)	18 (4.7)		83 (27.7)	97 (32.3)	71 (23.7)	49 (16.3)		7 (3.2)	29 (13.1)	43 (19.5)	142 (64.3)	
Support from other people													
Yes	1596 (97.7)	38 (2.3)	<0.001	279 (22.6)	369 (29,8)	288 (23.3)	301 (24.3)	0.010	25 (2.7)	108 (11.5)	133 (14.1)	676 (71.8)	0.137
No	138 (83.6)	27 (16.4)		39 (31.7)	38 (30.9)	31 (25.2)	15 (12.2)		0 (0)	10 (10.5)	20 (21.1)	65 (68.4)	

**Table 3:** Association between starting to think about breastfeeding (BF) and the duration of exclusive BF, *p* < 0.001. *N* = 1184.

Started Thinking About BF	Exclusive BF Duration in Months				
Trimester	<1 Month	1–3 Months	3–6 Months	>6 Months	Total
First	137 (18.7)	209 (28.5)	188 (25.6)	200 (27.2)	734
Second	38 (23.8)	50 (31.3)	34 (21.3)	38 (23.8)	160
Third	71 (32.7)	70 (32.3)	37 (17.1)	39 (18.0)	217
No intention to BF	26 (35.6)	19 (26.0)	21 (28.8)	7 (9.6)	73

**Table 4:** The proportion of all mothers (*N* = 1799) receiving breastfeeding advice from various health professionals and non-specialists.

	Number of Mothers Receiving Breastfeeding Advice *			
	Pre-Delivery		Post-Delivery	
	Encouraging *N* (%)	Discouraging *N* (%)	Encouraging *N* (%)	Discouraging *N* (%)
Health professionals	535 (29.4)	2 (0.1)	787 (38.4)	8 (0.4)
Lactation specialists	220 (12.1)	2 (0.1)	568 (27.7)	5 (0.3)
Mothers	437 (24)	7 (0.4)	310 (15.1)	11 (0.6)
In-laws	270 (14.9)	7 (0.4)	132 (6.4)	17 (0.9)
Spouses	262 (14.4)	5 (0.3)	165 (8)	11 (0.6)
Other relatives	61 (3.4)	13 (0.7)	55 (2.7)	22 (1.2)
Friends/others	33 (1.8)	7 (0.4)	34 (1.7)	13 (0.7)

* A mother may have received advice from one source or more, just as some mothers expressed not having received advice from anyone.

## Data Availability

The data that supports the findings of the current study are available from the corresponding author upon reasonable request.

## Abbreviations

aOR	Adjusted Odds Ratio
CI	Confidence Interval
EBF	Exclusive breastfeeding
SD	Standard Deviation
UAE	United Arab Emirates
WHO	World Health Organization
UK	United Kingdom
